# Achievements in and Challenges of Tuberculosis Control in South Korea

**DOI:** 10.3201/eid2111.141894

**Published:** 2015-11

**Authors:** Ji Han Kim, Jae-Joon Yim

**Affiliations:** Seoul National University College of Medicine, Seoul, South Korea

**Keywords:** tuberculosis, South Korea, public–private mix, control measures, bacteria, Mycobacterium tuberculosis, demographics

## Abstract

After the Korean War (1950–1953), nearly 6.5% of South Korea’s population had active tuberculosis (TB). In response, South Korea implemented the National Tuberculosis Program in 1962. From 1965 to 1995, the prevalence of bacteriologically confirmed pulmonary TB in South Korea decreased from 940 to 219 cases per 100,000 population. Astounding economic growth might have contributed to this result; however, TB incidence in South Korea remains the highest among high-income countries. The rate of decrease in TB incidence seems to have slowed over the past 15 years. A demographic shift toward an older population, many of whom have latent TB and various concurrent conditions, is challenging TB control efforts in South Korea. The increasing number of immigrants also plays a part in the prolonged battle against TB. A historical review of TB in South Korea provides an opportunity to understand national TB control efforts that are applicable to other parts of the world.

## Abstract 2

Despite astounding economic growth and TB control efforts, incidence remains the highest among high-income countries.

Despite the hardships resulting from the Korean War during the early 1950s, South Korea accomplished rapid economic growth and now enjoys one of the world’s largest economies and a high standard of living. In parallel with the economic prosperity, South Korea achieved admirable control of tuberculosis (TB) in the past half century. TB in South Korea is a classic example of how a country that was once one of the poorest in the world can drastically reduce its disease burden through national disease control efforts. However, TB incidence in South Korea remains the highest among high-income countries: as of 2013, TB incidence in South Korea was 7 times higher than the average incidence for member countries of the Organisation for Economic Co-operation and Development (*1*). To understand the interaction of disease with major social changes, we discuss how the economic transitions in South Korea during the 20th century shaped the country’s TB burden. We also address the major problems in TB control in South Korea today and current endeavors to control the disease.

## Part I. TB in South Korea during the 20th Century

Although historical accounts suggest that TB has existed on the Korean Peninsula for centuries, it is unclear when the causative agent, *Mycobacterium tuberculosis*, was first introduced to the region. TB, as described in modern medicine, did not appear in the official records of Korea until the turn of the 20th century. We therefore begin our discussion of TB at the beginning of the 20th century, during the time of Japanese colonization.

### The Colonial Period (1910–1945)

The Japanese Empire of the late 19th century strived for military growth. After victory in the war against Russia of 1905, the Empire of Japan integrated Korea as a protectorate, and in 1910 Korea was officially annexed as a colony. The Korean Peninsula had strategic importance because of its geographic location, and it also provided the empire with raw materials and a labor force. As industrialization in Korea began to expand, the population soared, especially in the cities. Population growth, overcrowding, and malnutrition increased, creating a more favorable environment for the spread of infectious agents.

In 1918, the colonial government implemented several strategies to control TB. To contain the spread of TB, the Japanese Governor-General of Korea installed bowls at various locations throughout cities and villages and ordered residents to spit their sputum into these containers. Patients who were found to have TB were isolated, and their possessions were cleaned. The colonial government also established health clinics equipped with x-ray devices to better diagnose TB (*2*). Western missionaries arrived in Korea, in cooperation with or independent from the colonial government, to help persons with TB. Dr. Sherwood Hall, a Methodist medical missionary from Canada, founded a sanatorium for TB care in 1928 (*3*).

**Figure 1:**
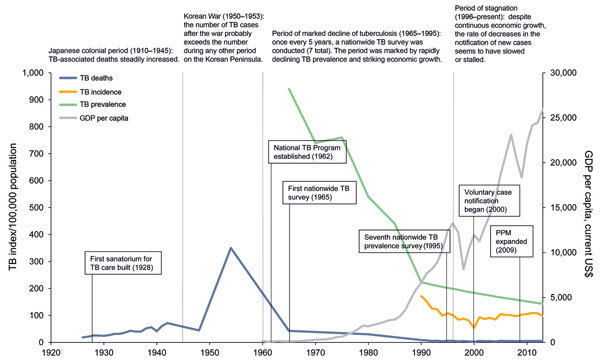
Number of tuberculosis (TB) cases per 100,000 population in South Korea, 1926–2013. Major periods are demarcated by dotted lines. Historical tuberculosis milestones for each period are briefly described. Notable tuberculosis control efforts are summarized in the boxes. GDP, gross domestic product; PPM, public–private mix. Sources: (*1*,*4*–*6*).

Limited data are available to accurately evaluate the epidemiologic status of TB in Korea during its colonial occupation by Japan. However, evidence suggests that TB death rates gradually increased during the second quarter of the 20th century. From 1926 to 1942, the Japanese imperial government expanded its political influence over Korea, China, and other parts of Southeast Asia. Records on deaths from TB during this period in Korea indicate that the death rate increased from 18.5 to 71.1 per 100,000 population (Figure 1) (*1*,*4*–*6*).

This increasing trend in TB-associated deaths was in contrast to the trend in most Western Europe countries during the same historical period. Most of these countries were recovering from the repercussions of World War I (1914–1918). For example, in England and Wales, the TB death rate reached its peak (135.8 deaths/100,000 population) when the war ended in 1918, but then the rate steadily decreased from 76.7 to 53.6 per 100,000 population during 1926–1942 (*7*,*8*). North America followed nearly the same trend as that of Western Europe. According to records of the city of New York, New York, USA, the death rate for TB decreased from 126 cases per 100,000 population in 1920 to 49 cases per 100,000 population in 1940 (*9*).

### The Korean War (1950–1953)

Korea gained its independence from Japan after World War II in 1945. The country was subsequently divided into North Korea and South Korea, which were protected by 2 competing superpowers, the Soviet Union and the United States, respectively. In 1950, the Korean War broke out and continued for 3 years. By the time an armistice agreement was signed in 1953, most social infrastructure in North and South Korea had been physically damaged or was dysfunctional.

After the Korean War, the number of deaths from TB increased drastically. Data collected in 1954 was used to estimate that 1.3 million of South Korea’s 20 million residents had active TB, and death rates ranged from 300 to 400 per 100,000 population (*5*). The last available data on TB before the Korean War were from 1942, when the death rate was 71.7 per 100,000 (*5*). A TB death rate that escalated 5-fold within a decade strongly points to the possibility that war was the strongest causative factor for the TB epidemic in South Korea. It has been well documented that armed conflicts increase deaths from TB for reasons that include malnutrition, overcrowding, and lack of access to health care (*7*,*10*).

By the 1950s, the first effective antimicrobial drugs against TB (i.e., streptomycin, para-aminosalicylic acid, and isoniazid) had been developed. However, it was difficult to deliver the drugs to TB patients in a war-torn country. It was not until 1955 that the Korean National Center for Tuberculosis began the first official distribution of anti-TB medications to 4,000 TB outpatients. The recommended regimen during this period was combined therapy with 2 or 3 of the drugs (*5*).

### Economic Growth (1965–1995)

During this period, South Korea experienced phenomenal economic development. This historical achievement is represented by an increase in gross domestic product (GDP) per capita from US $106 in 1965 to US $11,000 by 1995 (*6*). As the economy grew, so did the South Korean national capacity for TB control. The South Korean government established the National Tuberculosis Program 1962. A cornerstone of the program was to provide bacillus Calmette-Guérin (BCG) vaccination to the entire population. Since legislation of this plan, the infant BCG vaccination coverage rate increased dramatically, from 1% in 1965 to 79% by 1990 (*11*). National Health Insurance was enacted in 1963 and was extended to nearly the entire population by 1989, facilitating access to quality medical care. New medications for TB, ethambutol and rifampin, were introduced in the 1980s. The cure rate among TB patients treated in public health centers was 56% in 1983 but improved to 79% by 1994, and the treatment default rate decreased from 15% to 4% during the same period (*12*).

The Korean National Tuberculosis Association administered a nationwide survey every 5 years during 1965–1995. TB prevalence was determined on the basis of chest radiograph findings and sputum smear and culture results (Table 1). Results of the nationwide survey during this time were remarkable. From 1965 to 1995, the prevalence of active pulmonary TB, as determined on the basis of chest radiographs, was reduced from 5,065 to 1,032 cases per 100,000 population, an annual average decrease of 5.0%. The prevalence of smear-positive pulmonary TB dropped from 668 to 93 cases per 100,000 population, a decrease of 6.8% every year. The prevalence of latent infection (defined as a >10-mm induration reaction to 1 dose of Tuberculin PPD RT 23) in persons <30 years of age decreased from 55.9% in 1965 to 30.8% in 1995. In conjunction with this change, the annual risk for TB infection in the same period fell from 5.3% to 0.5%. Drug resistance, especially to isoniazid, dropped from 36.4% to 9.2%; overall resistance to >1 anti-TB drugs among new patients and those with a history of TB treatment decreased from 26.2% and 55.2% to 5.8% and 25.0%, respectively, during this 30-year period (*4*).

**Table 1:** Prevalence of pulmonary tuberculosis, South Korea, 1965–1995*

Confirmed cases	Prevalence, by year						
	1965, N = 20,117	1970, N = 26,314	1975, N = 27,090	1980, N = 23,319	1985, N = 39,704	1990, N = 48,976	1995, N = 64,713
All pulmonary cases, radiographically active							
No. cases							
Per 100,000 population	5,065	4,222	3,326	2,509	2158	1,842	1,032
Total	1,019	1,111	901	585	857	902	668
Patient sex							
M	614	650	551	374	534	577	445
F	405	461	350	211	323	325	223
Patient age, y							
5–19	223	296	215	80	103	131	30
20–34	219	203	175	130	218	153	90
35–49	266	273	203	153	203	190	134
50–64	206	193	192	134	185	243	222
>65	105	146	116	88	148	185	192
Bacteriologically confirmed cases, smear and/or culture positive							
No. cases							
Per 100,000 population	940	741	764	545	443	241	219
Total	189	195	207	127	176	118	142
Patient sex							
M	137	130	139	94	134	84	98
F	52	65	68	33	42	34	44
Patient age, y							
5–19	26	16	26	7	9	5	2
20–34	59	43	44	24	42	15	19
35–49	65	61	58	39	54	34	34
50–64	32	52	55	31	41	37	44
>65	7	23	24	26	30	27	43
Smear positive cases only							
No. cases							
Per 100,000 population	686	559	480	309	239	143	93
Total	138	147	130	72	95	70	60
Patient sex							
M	96	NA	92	57	77	53	39
F	42	NA	38	15	18	17	21
Patient age, y							
5–19	18	NA	10	5	3	2	1
20–34	45	NA	32	15	23	7	11
35–49	50	NA	42	25	37	26	11
50–64	18	NA	36	17	23	22	19
>65	7	NA	10	10	9	13	18

*Data are based on a nationwide tuberculosis survey that is conducted every 5 years (*4*). NA, not available.

Economic growth brought about not only greater national capacity for disease control but also changes in nutritional status of South Korea’s population. During this phase of a burgeoning economy, average body mass index increased, especially among children. In 1965, body mass index was 16.9 for children 12 years of age, and by 1995, it had reached 19.0. On average during this 30-year period, 12-year-old children increased in height by >10 cm and increased in weight by 9.5 kg (*13*). Because malnutrition and being underweight are major risk factors for TB, nutritional improvement linked to economic growth has served as a major contributing factor for successful TB outcomes in South Korea.

Other socioeconomic indicators have declined. From 1965 to 1995, the fertility rate declined from 5.0 to 1.6 births per mother, and the size of households decreased from 5.6 to 3.4 persons per household (*14*,*15*). These declines resulted from federal campaigns and family planning programs. During this same period, school class sizes decreased from 65.4 to 36.4 students per classroom. Such factors have led to less crowding at home and in school and the creation of living environments that are less favorable for disease transmission (*13*).

However, despite these improvements in South Korea, shortcomings remained with respect to TB control. Although the proportion of TB patients treated in the private sector increased from the 1980s to the 1990s, cure rates among private-sector patients in 1993 were generally lower than those for public health service patients (*12*,*16*). Inappropriate anti-TB regimens also contributed to poor treatment outcomes. In a survey conducted in 1993, only 11% of general practitioners prescribed the standard 6-month anti-TB treatment regimen, and as many as 86 different regimens were prescribed to 960 TB patients (*16*).

### Recent Decades (1996–Present)

A TB notification system was adopted in 1996 in South Korea, replacing the nationwide survey. According to government legislation, when a person receives a diagnosis of TB, the relevant medical staff is required to immediately notify the case to the public health center under jurisdiction. Annual reports on notified cases have been available since 2001. Meanwhile, the South Korean economy has shown steady growth, and GDP per capita has increased from US $12,250 in 1996 to US $22,590 in 2012 (*6*). By 2013, South Korea’s GDP was ranked 14th in the world.

Despite the growing economy and continuous national efforts to control TB, the disease maintains a strong foothold in South Korea, afflicting tens of thousands of new patients each year. The number of notified cases per 100,000 population has remained around 90 for more than a decade; the number of notified cases per 100,000 population reached 96.3 in 2001, peaked at 100.8 in 2011, and then decreased to 84.9 in 2014 (Table 2) (*17*). Some experts on TB in South Korea argue that TB incidence has, in fact, been stagnant; they base this argument on the possible undernotification of cases. According to a cross-sectional study of 37,820 TB patients identified from national health insurance claim data in 2008, only 21,611 (57.1%) cases were reported to the Korean Tuberculosis Surveillance System (*18*). To determine whether TB incidence is decreasing, more data are needed on the number of notified TB patients and the number of TB patients who received national health insurance benefits.

**Table 2:** Number of notified tuberculosis cases, South Korea, 2001–2014*

Type of notification	No. notified cases, by year													
	2001	2002	2003	2004	2005	2006	2007	2008	2009	2010	2011	2012	2013	2014
Cases per 100,000 population	96.3	89.4	83.8	86.1	96.5	94.7	92.8	89.4	95.3	96.4	100.8	98.4	89.6	84.9
Cases in public sector	15,728	13,003	11,810	10,851	9,680	9,018	7,558	7,315	7,079	5,463	4,461	3,779	3,269	2,994
Cases in private sector	18,395	19,007	18,877	20,652	25,589	26,343	27,152	26,842	28,766	30,842	35,096	35,766	32,820	31,875

*Source: (*17*).

**Figure 2:**
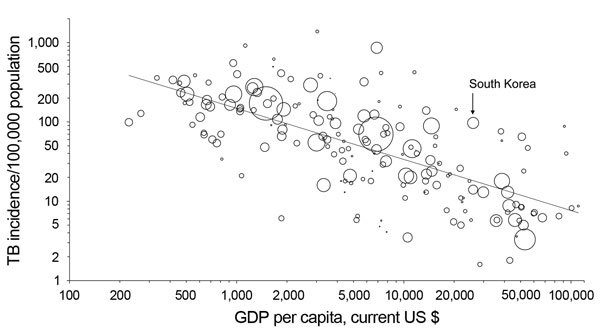
Relationship between per capita gross domestic product (GDP) and incidence of tuberculosis (TB), 2013. Each dot represents 1 country; South Korea is indicated. The third root of the population was used to determine the size of the circles, and the figure is drawn on a logarithmic scale. The line indicates the regression on the logarithm. The figure was adapted from (*19*) with permission from The European Respiratory Society. Updated data was derived from (*1*).

Notwithstanding such speculation, South Korea has a disproportionately high burden of TB compared with most high-income countries (Figure 2) (*19*). According to the World Health Organization (WHO), the incidence of TB in South Korea was 108 cases per 100,000 population in 2012, which is >7 times higher than the average for member countries of the Organisation for Economic Co-operation and Development. For example, the annual incidence of TB in the United States and Japan in 2012 was 3.6 and 19 cases per 100,000 population, respectively.

## Part II. Reasons for the High Burden of TB in South Korea

Recent data indicate that, among high-income countries, South Korea stands out in terms of TB burden, despite its astonishing rate of economic development. In this section, we attempt to delineate some of the most frequently discussed factors attributed to the tenacious character of TB in South Korea.

### High Prevalence of Latent TB Infection in the Elderly Population

**Figure 3:**
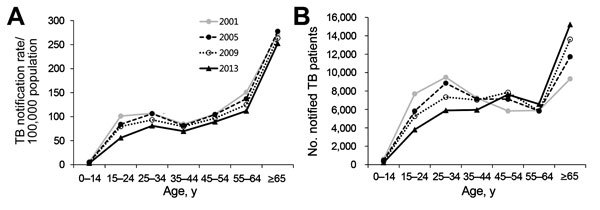
Rate of notified tuberculosis (TB) cases by age group and year, South Korea. A) Comparison of age-specific notified TB cases per 100,000 population between 2001 and 2013. In 2013, notification rates among all age groups were lower than those in 2001. B) Comparison of notified tuberculosis cases by patient age between 2001 and 2013. In 2013, there was a marked increase in the number of cases among persons 45–54 and >65 years of age. Figure adapted from (*21*).

An older population is at higher risk for TB because host immunity against *M. tuberculosis* wanes with aging (*20*). The elderly population in South Korea has steadily increased over the last 3 decades. Such demographic transition is reflected in the increased number and proportion of TB patients >65 years of age, rising from 9,322 (20.2%) in 2001 to 15,227 (33.6%) in 2013 (Figure 3) (*17*,*21*). Among South Korea residents >60 years of age without radiographic evidence of prior TB, 67.2% were found to have latent TB (*22*). TB was highly prevalent during the youth of most persons born during 1955–1963 in South Korea. This population group, with a possibly large number of latent infections, comprises 15% of the entire population of South Korea today. When this group officially becomes part of the elderly population in 2020, a resurgence of TB is possible, and this possibility casts a bleak outlook for TB control in South Korea.

### An Increasing Population with Diabetes

The increasing number of persons in South Korea with various concurrent conditions puts an additional burden on the country’s TB control program. The epidemiologic trend during the past few decades in South Korea generally moved away from infectious diseases and toward chronic diseases, such as diabetes, which puts patients at greater risk for developing TB. Diabetes mellitus is a common concurrent condition that shows a strong association with TB, particularly in TB-endemic developing countries with a rising prevalence of diabetes mellitus (*23*). TB is thought to induce glucose intolerance and make it more difficult for persons with diabetes to control blood glucose. This in turn causes them to be more susceptible to new infection, reactivation of latent infection, and aggravation of active disease (*24*). The prevalence of diabetes mellitus in South Korea has increased from 1.5% to 9.9% in the past 40 years (*25*), and a positive relationship between diabetes and TB has been documented in South Korea’s population (*26*).

### High Smoking Rate

Although the proportion of smokers in most industrialized countries is decreasing, many TB patients in South Korea still use tobacco. In 2012, the percentage of daily smokers among men and women >15 years of age was 37.6% and 5.8%, respectively (*27*). The number of male smokers is the highest among most industrialized countries. By comparison, the percentage of daily smokers among men >15 years of age in the United States and United Kingdom during 2012 was 15.9% and 20.3%, respectively. Smoking has been documented as a risk factor in terms of TB infection, disease, and death (*28*). In a prospective cohort study involving more than 1 million South Koreans, smoking was associated with increased TB incidence, recurrence, and death (*29*). For these reasons, smoking is likely to be responsible for the relatively high burden of TB in South Korea. The current Korean Guidelines for Tuberculosis recommend that persons who smoke and who receive a diagnosis of TB should be advised to stop smoking before treatment begins (*30*).

### Inadequate Patient Management

Thorough patient management during standard TB treatment has been difficult to achieve in South Korea. Directly observed therapy (DOT), short-course (DOTS), has been the hallmark of TB treatment for decades, and this program has been shown to be effective in various settings (*31*). Among the 5 elements of the DOTS strategy, DOT is an indispensable component; unfortunately, DOT is not practiced in South Korea. Furthermore, until recently, South Korea had no mandate for isolating smear-positive TB patients, even those who were noncompliant or who had multidrug-resistant disease, and most patients were treated in the outpatient setting. This lack of monitoring could have been potential barriers to the advancement of TB control.

The rationale for not adopting DOT in South Korea is that the treatment success rate in the public sector is ≈90%, which is higher than the DOTS target of 85% set by WHO (*32*). However, the proportion of TB patients receiving treatment in the private sector has increased to 90% in the past decade, and the treatment success rate for this sector was 75% in 2001 (10% below the WHO target). This low success rate suggests that poorly executed patient monitoring among private health care providers could have been the reason for the high treatment failure rates (*17*,*33*).

### Immigrants from High-Burden Countries

South Korea has witnessed a continuous influx of immigrants in recent years. The most common countries of origin are China, Mongolia, Pakistan, Philippines, Vietnam, and North Korea (*34*). The proportion of TB patients of foreign nationality has been increasing in recent years, from 0.3% in 2001 to 3.8% in 2013 (*17*). Because this increased percentage represents mostly documented immigrants, the actual number is thought to be much higher. Many of these immigrants have inadequate access to health care because of their socioeconomic and legal status. Furthermore, the number of North Korean defectors entering South Korea has increased in the past decade, and >1% are considered to have active TB at the time of entrance (*35*). Because of the diverse backgrounds and legal circumstances among this population at high risk for TB, adoption of a more rigorous screening program is necessary.

## Part III. Recent Efforts and Goals to Be Achieved

To overcome the challenges posed by TB, the South Korean National Tuberculosis Program has attempted to conduct rigorous monitoring activities in an effort to further reduce TB in the country. The “New 2020 Plan” set out by the Korean Centers for Disease Control aims to cut the incidence rate in half by 2020, preferably to <50 cases per 100,000 population. The 2 major arms of recent endeavors include expanded public–private mix collaboration and prompt outbreak investigations.

### Public–Private Mix Collaboration

In 2009, the government of South Korea initiated public–private mix collaboration for TB control based on WHO recommendations (*36*). Major components of the collaboration include strict monitoring of patients, investigation of close contacts, and financial support of patients hospitalized in the private sector with multidrug-resistant TB. Preliminary results of the program were successful; patients included in the public–private mix collaboration showed higher treatment success rates than controls (91.6% vs. 71.8%). The treatment default rate for patients in the collaboration program (6.6%) was lower than that for controls (22.9%), resulting in a better treatment outcome (*33*). In 2011, public–private mix collaboration was expanded to 97 health centers, in which 31,050 (92.4%) of 33,587 total patients were reported to have been treated under rigorous monitoring by nurses trained exclusively for TB patient management (*37*).

Public–private mix collaboration has a set of stipulations that must be fulfilled to meet the intended results. Key elements of successful collaboration are decentralization; transparency; mutual respect; working through consensus; private provider involvement at all levels, including the highest-level policymaking; continual dialogue; and quality assurance (*38*). To guarantee the success of public–private mix collaboration in South Korea, policymakers should regard frontline clinicians working in the private sector as partners in every aspect of the collaboration.

### Reinforcement of Outbreak Investigations

The Korean Centers for Disease Control strives to detect TB cases by screening anyone who is in frequent contact with a newly identified patient in various settings. If a notified patient with TB is a preschool child, school student, teacher, or member of the military or is living in an institutional facility, outbreak investigation must be enforced in accordance with the South Korean National Tuberculosis Program. When a school student is determined to have infectious TB, all students sharing the same classroom must be examined. In addition, if 2 school students in a class have had known TB in the previous 6 months, every student in the class is examined. If >3 students in a school have infectious TB, everyone in the school, including all students and teaching staff, should be examined. Chest radiographs and sputum microscopy are used to determine active TB. If results of these tests are normal, a tuberculin skin test is administered to screen for *M. tuberculosis* infection (>10 mm induration). If the skin test is reactive, an interferon-γ release assay is used to rule out false-reactive results, and treatment is offered to those determined to have TB (*39*). These guidelines have not been strictly followed but are now being strongly enforced. In 2013 alone, a total of 3,824 patients were notified from 3,265 institutional facilities and a total of 1,194 investigations were carried out, during which 1,476 possible contacts were tested for *M. tuberculosis* infection. Of these 1,476 persons, 939 were from schools, 142 were from military bases, 274 were from health care facilities, 64 were from occupational settings, and 57 were from other settings (*40*).

## Conclusion

The experiences of South Korea during the 20th century have been similar to those of many developing countries: independence from colonization, warfare, economic growth, and health conditions shifting toward chronic diseases as the population ages. Today, South Korea is a developed country, but it still has emerging problems with TB control, which largely stem from demographic transitions within the country. Despite the emerging challenges, South Korea’s National Tuberculosis Program can be seen as a successful model that can be applicable to other developing countries. The history of TB in South Korea illustrates that epidemiologic changes that occur along with the development of new drugs and diagnostic tools must be accompanied by changes in policymaking. Policymakers should pursue improved national TB programs to help meet the challenges of TB control in light of the continuously evolving global disease burden.
